# Management and Clinical Outcomes of Liver Hemangiomas: A Retrospective Comparative Study of Transcatheter Arterial Embolization and Surgery

**DOI:** 10.3390/medicina62091782

**Published:** 2026-09-16

**Authors:** Sefa Ergün, Fadime Kutluk, Rauf Hamid, Yasemin Pekmezci, Seyfullah Halit Karagöz, Mehmet Velidedeoğlu, Server Sezgin Uludağ, Ahmet Baş, Fatih Gülşen, Salih Pekmezci

**Affiliations:** 1 General Surgery Department, Cerrahpaşa Medical Faculty, Istanbul University-Cerrahpaşa, Istanbul 34854, Turkey; sefaergn@yahoo.com (S.E.); dryaseminpekmezci@gmail.com (Y.P.); m-veli@hotmail.com (M.V.); sszgn.uludag@gmail.com (S.S.U.); pekmezcisalih@gmail.com (S.P.); 2 Department of General Surgery, Kadıköy Florence Nightingale Hospital, Istanbul 34724, Turkey; drkutluk@hotmail.com; 3 Radiology Department, Cerrahpaşa Medical Faculty, Istanbul University-Cerrahpaşa, Istanbul 34854, Turkey; seyfullahhalitkaragoz@gmail.com (S.H.K.); ahmet.bas@iuc.edu.tr (A.B.); drfgulsen@gmail.com (F.G.)

**Keywords:** hepatic hemangioma, transarterial embolization, bleomycin

## Abstract

*Background and Objectives*: Hemangiomas are the most common benign tumors of the liver. Surgical options such as enucleation, segmentectomy, and hepatectomy are the primary treatment choices, though the morbidity and complication rates remain high. Transcatheter arterial embolization (TAE) has emerged as a safe and effective alternative treatment option that has gained popularity in recent years. *Materials and Methods*: We retrospectively evaluated data from patients who underwent either surgical intervention (*n* = 19) or transarterial embolization (62 lesions in 61 patients; analyses performed per treated lesion) for liver hemangioma between 2003 and 2021. Outcomes were assessed separately for each modality without a composite efficacy endpoint: clinical success (relief of the presenting symptoms, assessed in patients symptomatic at baseline), technical success and radiological response for embolization, treatment-related complications, length of hospital stay, and the need for reintervention. *Results*: There was no significant difference in the pretreatment size of the lesions between the TAE and surgery groups (median of 10.0 [IQR 7.8–13.9] versus 10.0 [IQR 9.0–17.0] cm; *p* = 0.100). The length of hospital stay was shorter in the TAE group (median of 1 versus 6 days; *p* < 0.001), although era-related differences in perioperative care may contribute to this difference, and follow-up was longer in surgically treated patients, reflecting the later adoption of embolization at our institution. Postoperative mortality did not occur in either group. In the embolization group, there were significant reductions in lesion size and volume and the complication rate was low, with no serious complications, supporting the safety profile of TAE. *Conclusions*: In this retrospective, non-randomized, single-center series, transarterial embolization was found to be a safe and effective minimally invasive treatment for liver hemangiomas and was associated with a shorter hospital stay. Because treatment allocation was not randomized, we cannot establish therapeutic equivalence between the two modalities, and the comparison should be regarded as descriptive; TAE represents an effective minimally invasive treatment option in appropriately selected patients and may be considered within a multidisciplinary treatment strategy, while surgery remains necessary in selected cases such as embolization failure, complicated lesion vascularization, or localization.

## 1. Introduction

Hemangiomas are the most common benign tumors of the liver. Supplied by the hepatic artery, they form blood-filled cavities lined with endothelial cells. The incidence of such lesions in the community ranges from 0.4% to 20%, and 80% occur in young females, predominantly between the ages of 30 and 50 [1,2]. Between 50% and 70% of liver hemangiomas (LHs) typically follow an asymptomatic course and are most commonly detected incidentally during radiological investigations. Those that cause symptoms or grow progressively may require treatment.

Symptoms depend on tumor size and location, with the most common being abdominal pain, dyspepsia, nausea, vomiting, loss of appetite, early satiety, and findings suggestive of compression. Although rare, ruptures and intra-abdominal bleeding have been recorded. The majority of LHs are small and commonly referred to as capillary hemangiomas. Only 10% exhibit progressive growth over time, although the reasons for this growth remain unclear [3]. The literature has no consensus on the size at which a hemangioma is considered giant; thresholds of 4, 5, and 10 cm have been proposed [2,4,5]. At our center, we use a 10 cm cut-off to consider hemangiomas to be giant. This threshold is chosen because pooled data indicate that the risk of moderate-to-severe adverse events after image-guided treatment rises significantly for lesions ≥10 cm, whereas 4–10 cm lesions generally follow a more benign course [4,5]. Giant hemangiomas reported in the literature seldom exceed a size of 20–35 cm [6].

Surgical treatment options such as enucleation, segmentectomy, and hepatectomy are typically safe and serve as the primary treatment choices. However, surgery may involve significant blood loss, long operation and hospitalization times, and perioperative complications [7,8]. In recent years, transcatheter arterial embolization (TAE) performed by interventional radiologists has emerged as a safe and growing alternative treatment option [4,9,10]. In the present study, the outcomes of patients diagnosed with liver hemangioma treated with either surgical interventions or transarterial embolization are evaluated.

## 2. Materials and Methods

In this retrospective study, data from patients who underwent either surgical intervention (*n* = 19) or transarterial embolization (62 lesions in 61 patients) for liver hemangiomas between 2003 and 2021 at a tertiary university center were evaluated, with imaging follow-up extending to August 2026. As we occasionally treated more than one hemangioma in the same patient, we performed all embolization analyses per treated lesion. The inclusion criteria were as follows: (1) giant hemangioma (>10 cm in the largest plane of measurement) and (2) hemangioma <10 cm significantly altering the patient’s quality of life (such as substantial pain and compression). The exclusion criteria included (1) patients under 18 years of age; (2) those diagnosed with hemangioma incidentally during diagnostic workup for hepatic surgery for tumors or metastases; and (3) those with a history of prior TAE procedures. The latter criterion referred to therapeutic embolization performed as definitive treatment before referral; planned preoperative devascularization embolization, typically performed within days before surgery to reduce operative blood loss rather than induce lesion regression, was not considered prior therapeutic TAE. We also excluded lesions with a final histopathological diagnosis that was not hemangioma (*n* = 2, echinococcal disease). This study was conducted in accordance with the Declaration of Helsinki and approved by the Ethics Committee of Istanbul University—Cerrahpaşa, Cerrahpaşa Medical Faculty. We obtained written informed consent from all patients to receive their treatment procedures before intervention; consent for inclusion in this retrospective analysis is outlined in the Informed Consent Statement.

**Figure 1:**
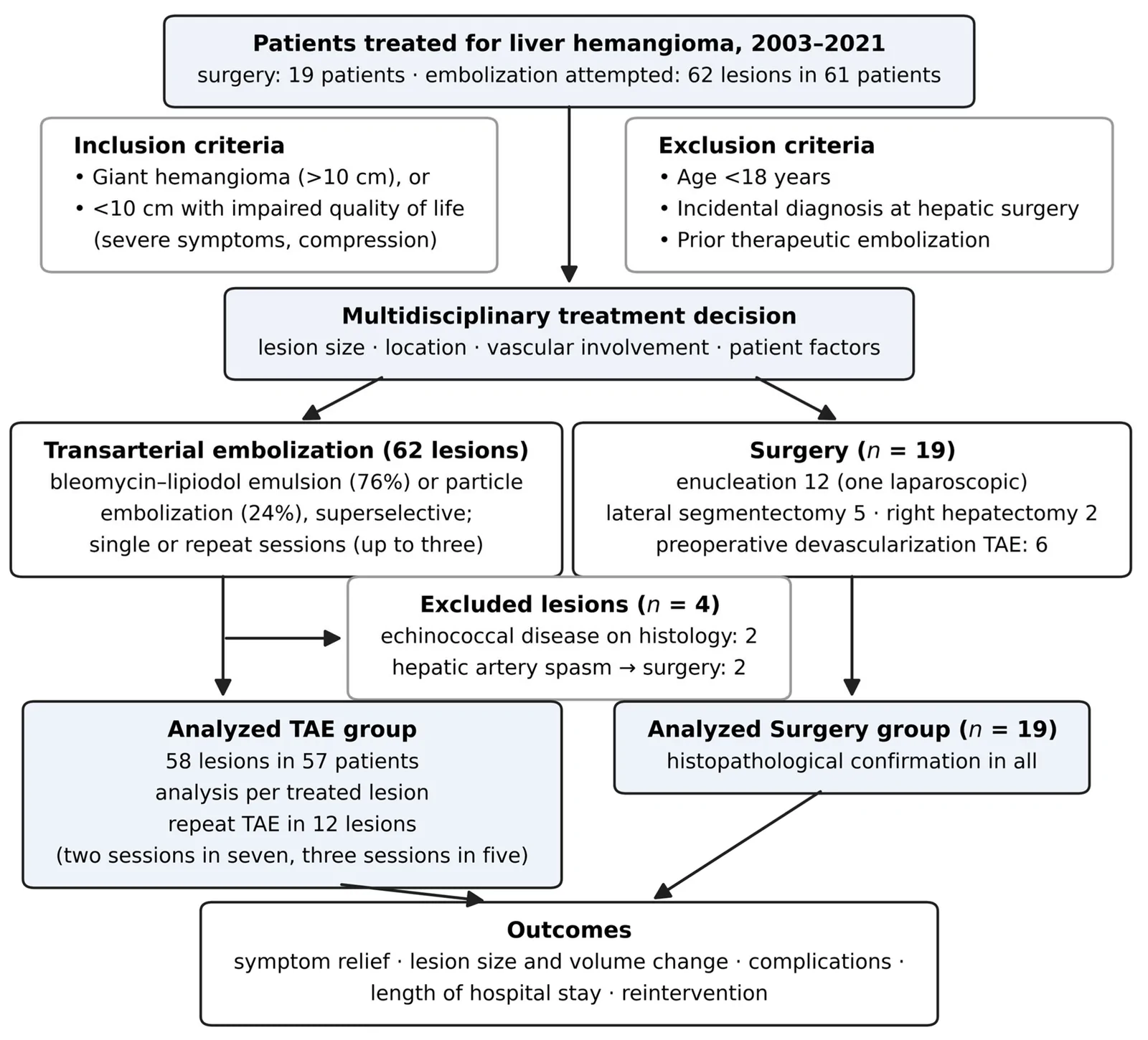
Treatment algorithm and flow of the study cohort. TAE: Transarterial embolization.

A multidisciplinary board of hepatobiliary surgeons and interventional radiologists determined treatment allocation on a case-by-case basis, considering lesion size, localization and relationship with major vascular structures, symptom severity, comorbidities, and patient preference (Figure 1). During the study period, TAE was generally offered as a first-line treatment for lesions suitable for superselective catheterization, whereas surgery was preferred for exophytic lesions that were causing compression or that required histopathological confirmation and for patients in whom embolization had failed or was technically unsuccessful.

We collected the patients’ demographic and clinical data, as well as data regarding radiological imaging findings, treatment methods, length of hospital stay, postoperative complications, and recurrence status, from radiological follow-ups, patient charts, and follow-up visits.

Gadolinium-enhanced magnetic resonance imaging (MRI) and triple-phase contrast-enhanced computed tomography (CT) were the primary diagnostic methods selected for treatment, whether surgically or interventionally. Abdominal ultrasonography (US) contributed to initial detection and clinical follow-up in a subset of patients, but it was never the sole imaging modality; all lesion size measurements were obtained from cross-sectional imaging. All resected specimens were verified via histopathologic examination. Lesion volume was calculated from three orthogonal diameters using the ellipsoid formula (V = 0.52 × a × b × c). Each treated lesion was measured and followed individually; when more than one hemangioma was treated in the same patient, the lesions were analyzed separately. A single radiologist specializing in abdominal and interventional radiology (F.G.) performed all measurements and was blinded to the clinical outcomes.

### 2.1. Surgical Resection Technique

The extent of surgical resection was determined based on the localization and size of the lesion, as well as its relationship with major vascular structures. Median and inverse-L abdominal incisions were the most common incision types. Enucleation was the preferred approach for patients with peripherally located lesions or those with insufficient residual hepatic volume. Conversely, regular segmentectomy or hepatectomy were favored for centrally located lesions or those involving major vascular structures; a representative perioperative image is provided in Supplementary Figure S1. The liver resection and enucleation treatments were conducted using an ultrasonic harmonic scalpel. Bipolar forceps were used for the electrocoagulation of small-diameter vessels, whereas larger vessels were ligated. The final diagnoses of patients undergoing surgical resection were confirmed via histopathological examination.

### 2.2. Transcatheter Arterial Embolization Procedure

Embolizations were performed by interventional radiologists (F.G. and A.B.) with 23 and 20 years of experience, respectively. Informed consent was obtained from all patients. Before the procedure, patients received varied pre-medication regimens tailored to each individual.

**Figure 2:**
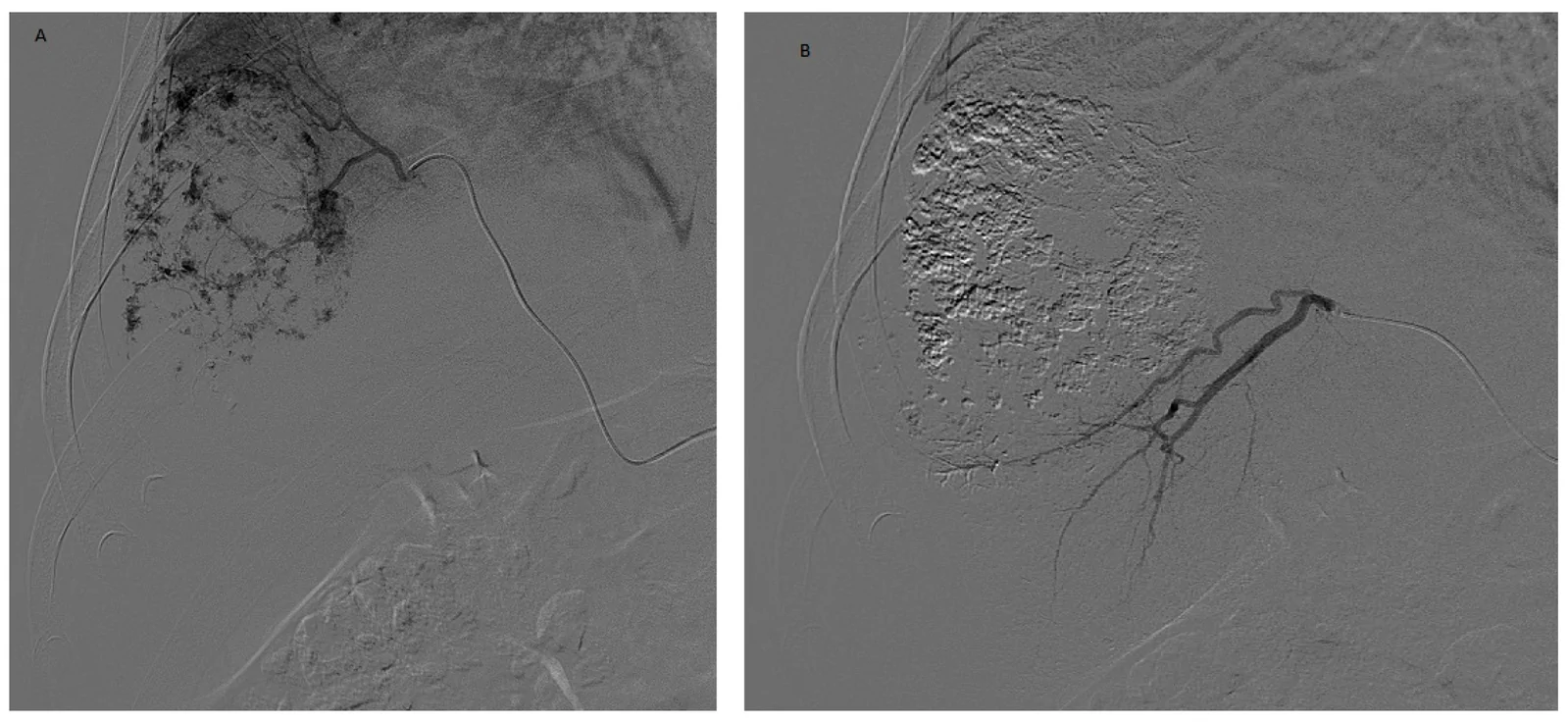
Angiography procedure (**A**) and subsequent application of bleomycin–lipiodol (**B**), distributed in the periphery of the hemangioma.

Using the Seldinger method under local anesthesia, a 5 French (F) vascular sheath was placed in the right common femoral artery. A diagnostic 5F pigtail catheter was navigated in the aorta to the level of the celiac artery origin. The aortic run revealed the main blood supply to the hemangioma. Afterward, a combination of a 5F USL catheter and a 2.7F microcatheter (Progreat, Terumo, Somerset, NJ, USA), with a 0.014-inch microguidewire, was used to catheterize the main feeder superselectively. Embolization was performed using a combination of lipiodol (Guerbet, Villepinte, France) with bleomycin sulfate (Bleosin-S, ONKO-Koçsel, Istanbul, Turkey). Drug dosing regimens for embolization were tailored to the size of the lesion: bleomycin sulfate (15 mg) + lipiodol (10 mL) + physiological saline (5 mL) for lesions < 10 cm and bleomycin sulfate (30 mg) + lipiodol (20 mL) + physiological saline (10 mL) for lesions > 10 cm. In 14 lesions (24.1%), embolization was performed with 300–500 µm calibrated spherical microspheres (Embosphere, Merit Medical, South Jordan, UT, USA) rather than the bleomycin–lipiodol emulsion, infused under fluoroscopic guidance until stasis of the feeding branch; polyvinyl alcohol (PVA) particles have also been used on occasion in our practice. The procedure proceeded until there was no hemangioma blush left or until considerable parenchymal or portal venous opacification was evident (Figure 2). Because no standardized recommendation exists for imaging surveillance after embolization of hepatic hemangiomas, follow-up imaging was not performed according to a fixed schedule. For each treated lesion, the first and the last available cross-sectional examinations were analyzed (contrast-enhanced CT or MRI, matched to the pretreatment modality whenever possible; ultrasonography was not used for measurements), and any cross-sectional study depicting the entire liver, including thoracic CT examinations extending below the diaphragm, was accepted. The first control examination was obtained at a median of 28 (IQR 11–69) months and the last at a median of 75 (IQR 45–98) months after embolization.

### 2.3. Outcomes

Because surgical resection removes the lesion whereas embolization induces regression of a lesion that remains in situ, outcomes were defined and reported separately for each treatment modality, and no composite efficacy endpoint was used for between-group comparison. Technical success was defined as complete devascularization of the lesion (absence of residual hemangioma blush) on the post-embolization angiographic run. Clinical success was defined as relief of the presenting symptoms and was assessed only in patients who were symptomatic at baseline. Radiological response, assessed for the TAE group, was defined as a reduction of more than 50% in lesion volume on follow-up imaging, the threshold most widely used in the literature and in recent meta-analyses [4,5]; a lesion that never reached this threshold was classified as an incomplete radiological response, representing primary radiological failure of TAE. Additional outcomes were treatment-related complications, length of hospital stay, and the need for reintervention (repeat embolization or surgery). Complications occurring within 30 days of treatment were recorded retrospectively from medical charts and structured patient interviews, and late treatment-related adverse events identified during follow-up were also recorded. Post-embolization syndrome was defined as self-limiting abdominal pain, fever, nausea, and/or leukocytosis without evidence of infection. Complications were graded with the Clavien–Dindo classification for surgery and the SIR adverse-event classification for TAE, and readmissions, hepatic dysfunction, biliary injury, infection, and access-site complications were recorded.

### 2.4. Statistical Analysis

Statistical analysis was performed with SPSS version 17.0 software. The propensity score, paired, and confidence-interval analyses were performed in Python 3.11 (SciPy and statsmodels). The suitability of the variables for normal distribution was examined with histogram graphs and the Kolmogorov–Smirnov test. Means with standard deviations are additionally retained for the descriptive baseline data in Table 1. Categorical variables were compared with Pearson’s chi-square test. Normally distributed continuous variables (age) were compared with Student’s t-test and are presented as the mean ± standard deviation, whereas non-normally distributed continuous variables were compared with the Mann–Whitney U test and are presented as the median with the interquartile range [IQR]. A *p*-value < 0.05 was considered statistically significant. Because treatment allocation was not randomized, a propensity score-based sensitivity analysis was additionally performed: the probability of undergoing surgery was estimated using a logistic regression model including age, sex, lesion size, and lesion multiplicity; the presenting symptom was initially considered as a covariate but was omitted because abdominal pain was nearly universal in the TAE group, which caused quasi-separation of the model. Inverse probability of treatment weighting (IPTW) was then applied. Covariate balance was assessed with standardized mean differences (SMDs), with values <0.10 indicating adequate balance. Because major determinants of treatment allocation (lesion location and morphology, vascular proximity, symptom severity, comorbidity burden, and treatment era) were not consistently available, weighting could not remove confounding by indication, and the propensity score-weighted analysis was reported as descriptive support only; the study should be regarded as a descriptive comparison of two institutional treatment pathways rather than a comparative-effectiveness analysis. Owing to the small number of outcome events, no multivariable outcome models were fitted to avoid overfitting. Fisher’s exact test was used instead of the chi-square test for categorical outcomes with small event counts. Exact *p*-values and 95% confidence intervals were reported for the main outcome comparisons, and paired pre- and post-embolization volumes were compared with the Wilcoxon signed-rank test. Analyses were performed on complete cases, without imputation of missing data. Because this retrospective study included all consecutive eligible patients, no a priori sample-size calculation was performed; this study was not designed to host an equivalence or non-inferiority comparison, no equivalence margin was prespecified, and, given the exploratory nature of the analyses, no adjustment for multiple comparisons was applied. In a treatment-strategy (intention-to-treat) sensitivity analysis, the two patients in whom embolization could not be completed because of vascular spasm and who subsequently underwent surgery were considered part of the TAE strategy group.

## 3. Results

Embolization was attempted for 62 lesions in 61 patients. Four lesions were excluded from the TAE analysis: two in which histopathological examination revealed echinococcal disease and two in which superselective catheterization could not be completed because of hepatic artery spasm, after which both patients underwent uneventful surgical resection (these two lesions are additionally analyzed as embolization failures in the treatment-strategy sensitivity analysis reported below). The final study population comprised 19 surgical patients and 58 embolized lesions in 57 patients. Of the patients in the surgery group, 12 (63.1%) were female and seven (36.9%) were male, with a median age of 53 years. The most common patient complaint was abdominal pain (63.2%), and the condition was identified incidentally in four patients (21%). The most frequently employed radiological methods were US (89.4%) followed by CT (84.2%) and MRI (42%). In the surgery group, 47.3% of the lesions were localized in the right lobe of the liver and 37% in the left lobe, while 15.7% of the cases had a bilateral localization. The size of hemangiomas ranged from 6 cm to 25 cm, with a mean diameter of 13.7 ± 6.4 cm and an average volume of 495.5 ± 254 cm^3^. Furthermore, 12 patients in the surgery group underwent enucleation, five underwent lateral segmentectomy, and two patients underwent right hepatectomy (Table 1). When the surgical cohort was examined by procedure type, the median length of hospital stay was similar after enucleation and after anatomical resection (6 days for both), and the single postoperative complication, an intra-abdominal abscess managed via percutaneous drainage (Clavien–Dindo grade IIIa), occurred after enucleation; the small subgroup sizes precluded formal statistical comparison.

**Figure 3:**
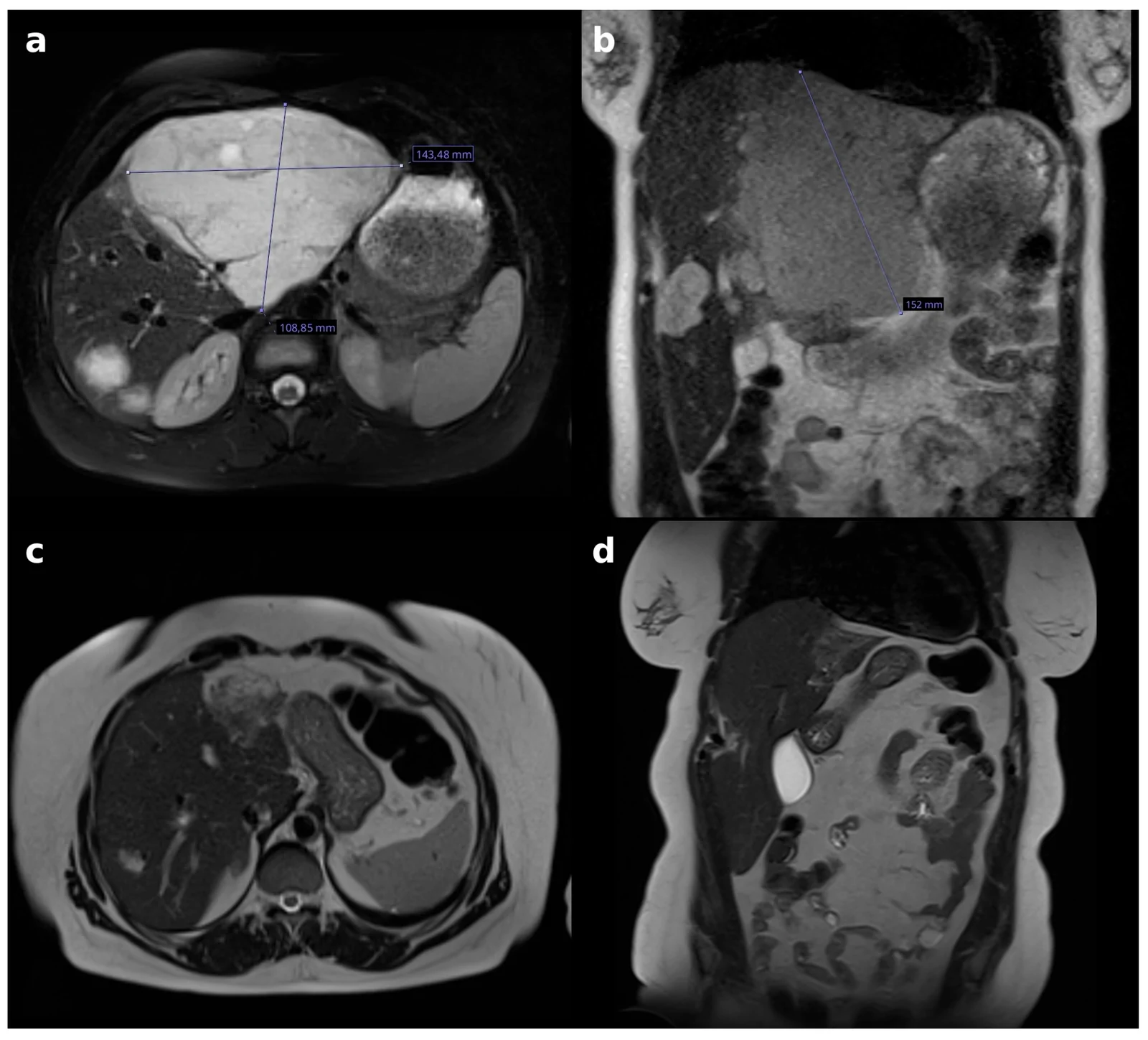
Pre- and post-embolization magnetic resonance imaging of a giant hemangioma of the left hepatic lobe. (**a**) Axial and (**b**) coronal T2-weighted images before embolization show a lesion measuring 15.2 × 14.3 × 10.9 cm. (**c**) Axial and (**d**) coronal images at the last follow-up, 85 months after embolization, show marked regression of the lesion to 5.5 × 4.3 × 3.9 cm (96% volume reduction).

**Figure 4:**
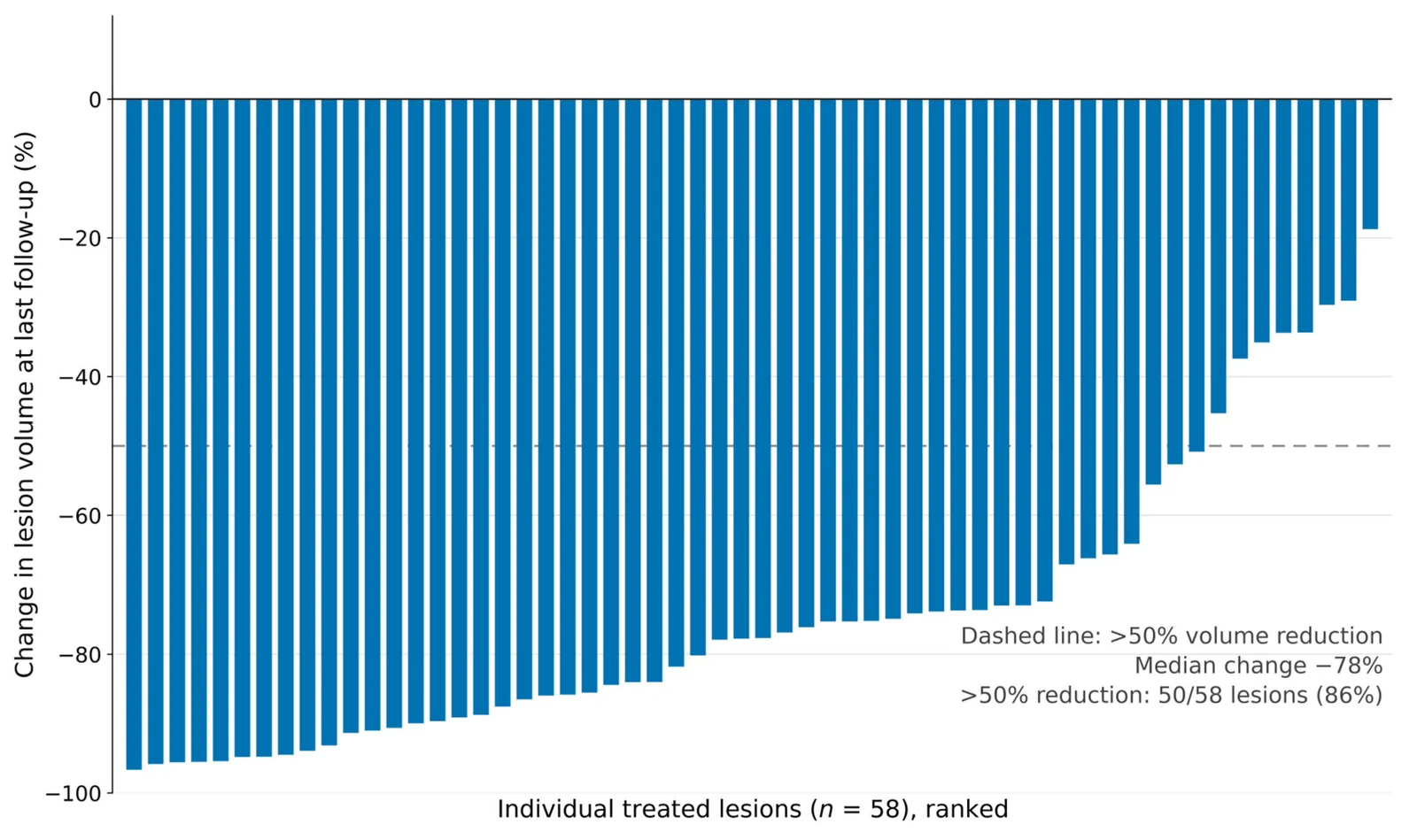
Waterfall plot of the percentage change in lesion volume between the pretreatment and the last follow-up imaging for each treated lesion (*n* = 58). The dashed line indicates the threshold of >50% volume reduction.

Of the 57 patients in the TAE group, 82.5% were female, and the median age was 50 years (mean 50.1 ± 8.5). The presenting complaint was abdominal pain for 57 of the 58 treated lesions (98.3%), and one lesion (1.7%) was detected incidentally; the cross-sectional methods used at diagnosis were CT in 56.9% and MRI in 51.7%, and abdominal US accompanied cross-sectional imaging in 12.1% of lesions, but it was never the sole diagnostic modality. The treated lesions were localized in the right lobe in 41 patients (70.7%), in the left lobe in 15 patients (25.9%), and in the caudate lobe in 2 patients (3.4%). The mean largest diameter of the treated lesions was 10.7 ± 4.0 cm (median 10.0, IQR 7.8–13.9; range 4.0–20.1 cm), and the mean pretreatment volume was 501 ± 533 cm^3^ (median 292, IQR 123–705 cm^3^); this decreased to 124 ± 200 cm^3^ (median 48, IQR 20–149 cm^3^) at the last imaging follow-up. The reduction in lesion volume between the pretreatment and the last follow-up examination was significant (Wilcoxon signed-rank test, *p* < 0.001), with a median per-lesion volume change of −77.7% (95% CI: −85.0% to −74.4%). At the last available imaging, the mean per-lesion reduction was 38.6% in the largest diameter and 74.4% in volume (Figure 3); radiological success (>50% volume reduction) was achieved for 45 of 58 lesions (77.6%) at the first control examination and for 50 of 58 lesions (86.2%) at the last follow-up. The per-lesion volumetric changes are shown in Figure 4. Bleomycin–lipiodol emulsion was the embolizing agent for 44 lesions (75.9%), and spherical particle embolization (300–500 µm calibrated microspheres; Embosphere, Merit Medical, South Jordan, UT, USA) was used for 14 lesions (24.1%). Repeat embolization was performed for 12 lesions (20.7%; two sessions in seven and three sessions in five). Post-embolization syndrome occurred in three patients (5.3%) and was managed symptomatically, and one patient (1.8%) developed recurrent episodes of cholangitis after embolization, which were managed conservatively; no other major complications were recorded.

There was no significant difference in the pretreatment size of the lesions between the TAE and surgery groups (median 10.0 [IQR 7.8–13.9] versus 10.0 [IQR 9.0–17.0] cm; *p* = 0.100). The length of hospital stay was significantly shorter in the TAE group than in the surgery group (median of 1 [IQR 1–1] versus 6 [IQR 5–7.5] days; *p* < 0.001) (Table 2). Because most resections were performed in the earlier part of the study period, part of this difference may reflect era-related changes in perioperative care and discharge practice rather than the treatment modality itself. The duration of follow-up was also significantly shorter in the TAE group (median of 75 [IQR 45–98] versus 120 [IQR 92–132] months; *p* = 0.008), which reflects the later adoption of embolization at our institution rather than a feature of the treatments themselves. No postoperative mortality occurred in either group. Treatment-related complications occurred in 1 of 19 surgical patients (5.3%) and 4 of 57 TAE patients (7.0%) (risk difference of −1.8%; 95% CI −12.4% to 18.1%; Fisher’s exact test, *p* = 1.000).

Among the 15 surgical patients who were symptomatic at baseline, the presenting symptoms resolved in all; the four incidentally detected lesions were not included in this denominator. In the TAE group, technical success was achieved for 96.7% of the attempted hemangioma lesions, and radiological response was achieved for 86.2% at the last follow-up; a standardized post-treatment symptom assessment was not available for the embolization group (see Section 4). In the propensity score-weighted sensitivity analysis, inverse probability weighting substantially improved the balance of baseline covariates between the groups (SMD <0.10 for age, lesion size, and lesion multiplicity), and the findings of the unadjusted analysis remained unchanged: the weighted mean length of hospital stay was shorter after TAE (1.0 versus 7.1 days); given the small number of events, complications were compared without weighting. These weighted estimates should be interpreted as descriptive support only, because weighting cannot remove treatment-selection bias arising from unmeasured allocation determinants.

When the analysis was performed according to the initial treatment strategy, embolization was technically successful for 58 of the 60 attempted hemangioma lesions (96.7%); for the remaining two lesions (3.3%), superselective catheterization could not be completed because of hepatic artery spasm, and both patients underwent surgical resection without complications. Within the surgery group, outcomes were similar in patients who received preoperative devascularization embolization (*n* = 6; median hospital stay 5.5 days, no complications) and in those treated with surgery alone (*n* = 13; median hospital stay 6 days, one complication). Preoperative devascularization embolization in these six patients served a purpose distinct from that of therapeutic TAE: it was performed to reduce the risk of intraoperative bleeding, and, because resection followed within days, it could not induce the gradual lesion regression that develops over months after therapeutic embolization; the outcomes of the surgical group therefore reflect surgical treatment itself rather than a combined treatment strategy. Reflecting the progressive shift in institutional practice, most surgical resections were performed in the earlier part of the study period, whereas the majority of embolization procedures were performed after 2014 (16 of 19 resections before 2014, versus 52 of 58 embolized lesions from 2014 onward).

With regard to reintervention, repeat embolization was performed for 12 of 58 lesions (20.7%), and no patient required salvage surgery after a technically successful embolization. At the final follow-up, eight lesions (13.8%) showed an incomplete radiological response (primary radiological failure of TAE), never having reached the 50% volume-reduction threshold. During follow-up, no treated lesion in either group showed relevant regrowth (no embolized lesion regrew beyond 50% of its original volume, and no local recurrence was observed after resection), and no retreatment for regrowth was required. The treatment indication was symptomatic disease for 57 of 58 embolized lesions and 15 of 19 surgical patients; incidentally detected giant lesions accounted for one embolized lesion (13.8 cm) and three surgical patients (10–16 cm), and one surgical patient with a 6 cm incidental lesion was treated owing to their preference.

## 4. Discussion

Although the etiology of liver hemangiomas is not yet fully understood, genetic predisposition and exposure to high levels of estrogen and progesterone in women have been implicated. The higher incidence in women suggests that there is possibly an association with pregnancy and the use of oral contraceptives [1]. The localization and size of the hemangioma primarily determine the need for treatment. Symptoms are related to increases in the size of the lesion, leading to the formation of infarction zones, the stretching of the liver capsule, and the compression of neighboring organs [1,2]. Anemia, thrombocytopenia, heart failure, and rupture can occur as a result of the progressive growth of the lesion [2,11].

Ultrasound is the primary diagnostic method, revealing hyperechoic, homogeneous nodules with well-defined margins, although CT and MRI offer greater sensitivity and specificity [2,12]. On CT scans, hemangiomas typically appear as uniform, clearly defined low-density lesions, and peripheral discontinuous progressive nodular contrast enhancement is observed following the administration of contrast material. On T1-weighted MRI sequences, hemangiomas exhibit a uniform low signal with well-defined edges, whereas on T2-weighted sequences, they typically display a distinctly high signal and the characteristic hemangioma “light bulb sign”, together with a central cleft-like area in larger lesions [2,12].

When weighing treatment options, factors such as the patient’s age, gender, and occupation should be considered, as well as their symptoms and the size and localization of the lesion. Treatment indications are broader in selected situations, such as in patients with high-impact occupations (e.g., boxing and football) with an increased risk of trauma, and young women of childbearing age, given the presumed hormone sensitivity of these lesions [1]. Although surgery was previously the mainstay of treatment, the risk of postoperative complications and hepatic parenchymal insufficiency, together with technological advances, has made minimally invasive interventional techniques increasingly preferred, including transarterial embolization, ablation, and percutaneous sclerotherapy [4,10].

The selection of the surgical approach depends on the location of the lesion. Enucleation is adequate for superficial hemangiomas situated peripherally, while deeper hemangiomas adjacent to major vascular structures and bile ducts may require resection (such as segmentectomy or hepatectomy) or orthotopic liver transplantation [13,14]. Potential complications following surgery include hemorrhage, bile leakage, intra-abdominal collections, and wound site infection [14]. Technological advances have enabled the application of laparoscopic or robotic-assisted hepatic resections in eligible patients, resulting in reduced complications and improved postoperative recovery [15].

Studies comparing resection and enucleation techniques in the literature recommend prioritizing the enucleation technique due to its lower risk of mortality and morbidity, as well as the preservation of liver parenchyma [16,17,18]. In Jiang et al.’s meta-analysis, enucleation was associated with a significantly shorter operative time, less intraoperative blood loss, fewer postoperative complications, and a shorter hospital stay than hepatectomy [17]. Of the 19 patients who underwent surgery in the present study, 12 underwent enucleation and seven underwent resection; one of these procedures, performed for a lesion localized in the left lateral segment of the liver, was completed laparoscopically. The symptoms in all patients subsided following surgical treatment.

Hemangiomas are suitable for embolization using the TAE technique due to their vascular supply from the hepatic artery. The embolizing agents employed in this study include polyvinyl alcohol, iodized oil, gelatin sponges, bleomycin, and ethanol [19]. Our clinic uses a mixture of bleomycin and lipiodol as the embolizing agent, in which bleomycin exerts antineoplastic activity by inhibiting DNA synthesis, while lipiodol serves as a carrier for delivering bleomycin to the target [7,20]. The combination of bleomycin and lipiodol functions not only as an embolizing agent but also as a chronic sclerosing agent, promoting fibrosis [20]. The mixture diminishes blood supply to the hemangioma, resulting in shrinkage of the lesion. In the treatment of hepatic hemangiomas, the bleomycin and lipiodol mixture operates by destroying endothelial cells, which prompts microthrombus formation in the sinuses, ultimately resulting in tumor mass atrophy and fibrosis [21]. Conversely, when combined with lipiodol, bleomycin is swiftly flushed out before it can harm the normal vessels. In a comprehensive meta-analysis of transarterial embolization reported by Torkian et al., symptomatic improvement occurred in 63.3–100% of cases (pooled clinical response rate, 98%), with a significant pooled reduction in hemangioma size and no mortality attributable to embolization [19]. In the present study, the embolization procedure was technically successful for 96.7% of the attempted hemangioma lesions. Our results are consistent with recent pooled evidence. In a meta-analysis of 2617 patients treated with image-guided therapies for giant hepatic hemangiomas, Günkan et al. reported pooled technical and clinical success rates of 99.9%, with moderate-to-severe adverse events in only 1.6% of patients and the lowest adverse-event rate observed for transarterial chemoembolization [4]. In a systematic review of transarterial (chemo-)embolization comprising 1284 patients, a reduction in tumor size in approximately 90% of patients, symptomatic improvement in 98.5%, grade 3 complications in 2.9%, and need for subsequent surgery in only 2.7% were found [22]. A recent meta-analysis comparing transarterial chemoembolization with percutaneous sclerotherapy of bleomycin reported comparable radiological success (approximately 82%) and rare major complications for both administration routes [5], and a durable medium- and long-term reduction in lesion size after bleomycin–lipiodol embolization was demonstrated in a series of 241 patients followed for up to five years [23]. Consistent with this durability, no treated lesion in our series showed relevant regrowth during follow-up in either group. The mean per-lesion reductions of 38.6% in diameter and 74.4% in volume observed in the present study, together with the low complication rate and the absence of major complications, are in keeping with these pooled estimates. A structured comparison of the present cohort with recent series and pooled analyses is provided in Table 3. While TAE can be considered a primary treatment method, it can also be employed in preoperative preparation to devascularize the lesion, minimizing intraoperative blood loss during surgery [1]. In the present study, six of the 19 patients who underwent surgery also received preoperative embolization.

The most frequent side effect associated with embolization is post-embolization syndrome, characterized by symptoms such as pain, fever, and leukocytosis. Furthermore, some patients may develop severe complications such as biloma, sclerosing cholangitis, hepatic abscess, hepatic insufficiency, and sepsis [24,25]. To prevent these undesirable complications and avoid gallbladder infarction, the embolization procedure should entail a slow infusion and the selective occlusion of the hemangioma [25]. In the present series, post-embolization syndrome occurred in three patients (5.3%) and recurrent cholangitis occurred in one patient (1.8%), with all cases managed conservatively.

At our clinic, when treatment is indicated according to the criteria described in the Section 2, embolization is generally the preferred initial approach when the lesion is anatomically suitable for superselective catheterization. A selective embolization procedure is performed in eligible patients, and surgical treatment is performed if the embolization procedure proves unsuccessful, particularly for lesions with peripheral or exophytic localizations causing compression. This study has several limitations that should be acknowledged. First, its retrospective, non-randomized design entails an inherent risk of treatment-selection bias: patients were allocated to surgery or TAE via the making of a multidisciplinary decision rather than at random, so the two groups cannot be assumed to be prognostically comparable, and the propensity score-weighted analysis cannot fully eliminate confounding by unmeasured factors. Second, the surgical cohort was small and heterogeneous, comprising enucleation, segmentectomy, and hepatectomy, and the numbers in the subgroups were too small for a statistical comparison to be made. Third, the follow-up duration differed significantly between the groups (median of 120 versus 75 months), which may bias comparisons of long-term outcomes and complications. In addition, the 19-year inclusion window inevitably encompasses substantial evolution of surgical technique, embolization materials, imaging standards, and periprocedural care, and treatment allocation shifted toward embolization over time, so calendar-time confounding cannot be excluded. Embolization outcomes were analyzed per treated lesion, so a small number of patients contributed more than one lesion; imaging follow-up was not protocolized, and the intervals between examinations were heterogeneous. Fourth, operative time, estimated blood loss, and transfusion requirements were not systematically recorded in the retrospective charts and could therefore not be analyzed. Comorbidity burden, ASA class, body mass index, previous abdominal surgery, detailed lesion morphology (central versus peripheral location, exophytic growth, proximity to major vessels or bile ducts), and standardized symptom-severity scores were likewise not uniformly available; quality of life, time to return to normal activity, and patient satisfaction were not assessed with validated instruments, and a standardized post-treatment symptom assessment was not available for the embolization group. Finally, this study reflects a single-center experience with a limited number of outcome events, which precluded multivariable outcome modeling. Accordingly, our findings should be interpreted as descriptive of institutional practice rather than as evidence of therapeutic equivalence between the two treatment modalities.

## 5. Conclusions

In this retrospective single-center cohort, transarterial embolization proved to be a safe and effective treatment for liver hemangiomas, providing significant lesion regression and symptom relief with a low complication rate and a shorter hospital stay than surgery, although era-related changes in perioperative practice may contribute to the latter difference. In line with the literature, the treatment approach at our clinic has progressively shifted towards minimally invasive embolization over the years. Nevertheless, given the non-randomized design, the potential for treatment-selection bias, and the unequal follow-up between the groups in this study, our data provide a descriptive comparison of two institutional treatment pathways and do not establish therapeutic equivalence or comparative effectiveness. TAE represents an effective minimally invasive treatment option in appropriately selected patients and may be considered within a multidisciplinary treatment strategy, while surgery retains its well-defined role in cases of embolization failure, complicated lesion localization, or diagnostic uncertainty. Prospective comparative studies are needed to confirm these findings.

## Figures and Tables

**Table 1:** Radiological and clinical characteristics of liver hemangiomas according to their groups. TAE values are reported per treated lesion (58 lesions in 57 patients).

	Surgery *n* (%)/Mean ± s.d.	TAE *n* (%)/Mean ± s.d.	Total *n* (%)/Mean ± s.d.
Size (cm)	13.7 ± 6.4	10.7 ± 4.0	11.4 ± 4.8
Volume (cm^3^)	495.5 ± 254.0	501 ± 533	500 ± 478
Lesion number			
Single lesion	16 (84.3)	29 (50.0)	45 (58.4)
Multiple lesions	3 (15.7)	29 (50.0)	32 (41.6)
Location			
Right	9 (47.3)	41 (70.7)	50 (64.9)
Left	7 (37)	15 (25.9)	22 (28.6)
Bilateral	3 (15.7)	–	3 (3.9)
Caudate	–	2 (3.4)	2 (2.6)
Symptoms			
Abdominal pain	12 (63.2)	57 (98.3)	69 (89.6)
Early satiety	3 (15.7)	–	3 (3.9)
Incidental	4 (21)	1 (1.7)	5 (6.5)
Diagnostic technique			
US	17 (89.4)	7 (12.1)	24 (31.2)
CT	16 (84.2)	33 (56.9)	49 (63.6)
MRI	8 (42)	30 (51.7)	38 (49.4)

TAE: transarterial embolization; US: ultrasound; CT: computed tomography; MRI: magnetic resonance imaging; s.d.: standard deviation.

**Table 2:** Comparison of demographic, clinical, and postoperative data between the two groups.

Variables	Surgery *n* (%)/Median [IQR]	TAE *n* (%)/Median [IQR]	*p*
Gender (male/female)	7/12 (36.9/63.1)	10/47 (17.5/82.5)	0.112 ^a^
Age (years)	53.1 ± 8.9	50.1 ± 8.5	0.193 ^d^
Size (cm)	10 [9–17]	10.0 [7.8–13.9]	0.100 ^b^
Hemoglobin (g/dL)	12.0 [11.0–13.5]	11.6 [10.2–12.8]	0.257 ^b^
Length of hospital stay (days)	6 [5–7.5]	1 [1–1]	<0.001 ^b^
Postoperative complication	1 (5.3)	4 (7.0)	1.000 ^c^
Follow-up (months)	120 [92–132]	75 [45–98]	0.008 ^b^

^a^ Chi-square test; ^b^ Mann–Whitney U test; ^c^ Fisher’s exact test. Continuous variables are shown as median [interquartile range], except age (normally distributed), which is shown as mean ± standard deviation. ^d^ Student’s *t*-test. TAE values are reported per treated lesion (*n* = 58; sex and age per patient).

**Table 3:** Recent series and pooled analyses of transarterial and surgical treatment of hepatic hemangiomas.

Study [Ref]	Design	Patients, *n*	Treatment	Symptomatic Response	Radiological Response	Major Complications/Reintervention
Torkian et al., 2021 [19]	SR + MA, 21 studies	1450	TAE (lipiodol-based emulsions or PVA)	Pooled symptom response 98%	Pooled diameter reduction −4.4 cm	No fatal complications; PES most frequent AE
Furumaya et al., 2019 [22]	SR, 18 cohorts	1284	TAE/lipiodolization	Symptom improvement 98.5%	Size reduction in 89.9% (9.8 → 4.0 cm)	Grade ≥ 3 in 2.9%; subsequent surgery in 2.7%
Günkan et al., 2025 [4]	SR + MA, 28 studies	2617	Image-guided (TACE, RF and MW ablation, PS)	Clinical success 99.9%	Radiological success 85.7%	Grade 2–4 AEs 1.6% (10.6% for lesions ≥ 10 cm)
Elek et al., 2025 [5]	SR + MA, 17 studies	1692	TACE vs. percutaneous bleomycin sclerotherapy	Clinical success 99.9% vs. 89.7%	Radiological success ≈ 82% for both routes	Major complications 0.27%
Yuan et al., 2022 [23]	Retrospective cohort	241	TAE, lipiodol–bleomycin	Symptom improvement 100%	Diameter reduction > 50% in 86.5% at 5 years	No serious complications reported
Zhao et al., 2024 [21]	Retrospective, three centers	102	TAE, bleomycin–lipiodol + gelatin sponge	NR	Volume 412.6 → 102.0 cm^3^; ≥50% volume reduction in 80.7%	No severe complications or death
Abdel Wahab et al., 2018 [14]	Retrospective surgical series	144	Surgery (enucleation 63.9%, anatomical resection 36.1%)	NR	Complete lesion removal (inherent to resection)	Low morbidity and mortality; enucleation and resection outcomes similar; blood loss and operative time higher for lesions > 10 cm
Farhat et al., 2021 [8]	Retrospective surgical series	12	Surgery (hepatectomy, lobectomy, enucleation)	All surviving patients asymptomatic at 55 months	Complete lesion removal (inherent to resection)	Morbidity 4/12, one death; median blood loss 870 mL; stay of 5.3 days
Present study	Retrospective comparative	19 surgery/58 TAE lesions (57 patients)	TAE, bleomycin–lipiodol vs. surgery	Symptom relief in all surgical patients; TAE technical success 96.7%	TAE: per-lesion diameter −38.6%, volume −74.4% (median −77.7%)	One surgical complication (CD IIIa); three with PES and one with cholangitis after TAE; repeat TAE in 20.7% of lesions

MA: meta-analysis; SR: systematic review; TAE: transarterial embolization; TACE: transarterial chemoembolization; RF: radiofrequency; MW: microwave; PS: percutaneous sclerotherapy; PVA: polyvinyl alcohol; PES: post-embolization syndrome; AE: adverse event; CD: Clavien–Dindo; NR: not reported.

## Data Availability

The data presented in this study are available on request from the corresponding author.

## Supplementary Materials

The following supporting information can be downloaded at: https://www.mdpi.com/article/10.3390/medicina62091782/s1, Figure S1: Intraoperative image of a giant hemangioma.
