# Supplementary material for: Management and Clinical Outcomes of Liver Hemangiomas: A Retrospective Comparative Study of Transcatheter Arterial Embolization and Surgery
Source: Medicina (Kaunas). 2026 Sep 16;62(9):1782. doi: 10.3390/medicina62091782 (PMC13609660; doi:10.3390/medicina62091782)

Figure S1: Intraoperative image of a giant hemangioma

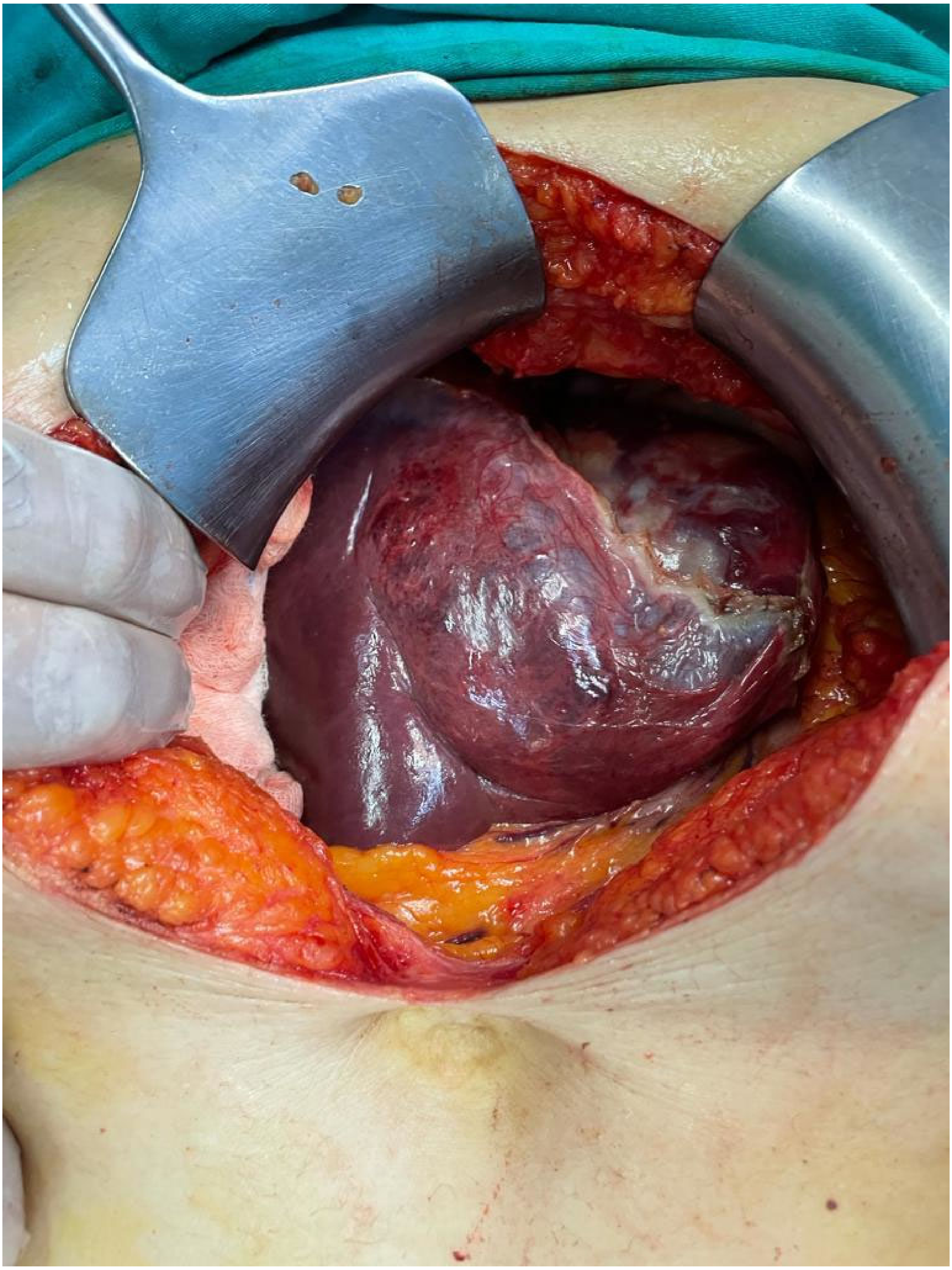

Supplement: Supplementary file 1 [file medicina-62-01782-s001.zip › medicina-4427383-supplementary.pdf]
